# Is artificial intelligence for medical professionals serving the patients? Protocol for a systematic review on patient-relevant benefits and harms of algorithmic decision-making

**DOI:** 10.1186/s13643-024-02646-6

**Published:** 2024-09-06

**Authors:** Christoph Wilhelm, Anke Steckelberg, Felix G. Rebitschek

**Affiliations:** 1 Institute of Health and Nursing Sciences, Martin Luther University Halle-Wittenberg, Magdeburger Str. 8, Halle, 06112 Germany; 2 Harding Center for Risk Literacy, Faculty of Health Sciences Brandenburg, University of Potsdam, Virchowstr. 2, Potsdam, 14482 Germany; 3 Max Planck Institute for Human Development, Lentzeallee 94, Berlin, 14195 Germany

**Keywords:** Algorithmic decision-making, ADM, Artificial intelligence, Patient relevant, Healthcare professionals, Decision support

## Abstract

**Background:**

Algorithmic decision-making (ADM) utilises algorithms to collect and process data and develop models to make or support decisions. Advances in artificial intelligence (AI) have led to the development of support systems that can be superior to medical professionals without AI support in certain tasks. However, whether patients can benefit from this remains unclear. The aim of this systematic review is to assess the current evidence on patient-relevant benefits and harms, such as improved survival rates and reduced treatment-related complications, when healthcare professionals use ADM systems (developed using or working with AI) compared to healthcare professionals without AI-related ADM (standard care)—regardless of the clinical issues.

**Methods:**

Following the PRISMA statement, MEDLINE and PubMed (via PubMed), Embase (via Elsevier) and IEEE Xplore will be searched using English free text terms in title/abstract, Medical Subject Headings (MeSH) terms and Embase Subject Headings (Emtree fields). Additional studies will be identified by contacting authors of included studies and through reference lists of included studies. Grey literature searches will be conducted in Google Scholar. Risk of bias will be assessed by using Cochrane’s RoB 2 for randomised trials and ROBINS-I for non-randomised trials. Transparent reporting of the included studies will be assessed using the CONSORT-AI extension statement. Two researchers will screen, assess and extract from the studies independently, with a third in case of conflicts that cannot be resolved by discussion.

**Discussion:**

It is expected that there will be a substantial shortage of suitable studies that compare healthcare professionals with and without ADM systems concerning patient-relevant endpoints. This can be attributed to the prioritisation of technical quality criteria and, in some cases, clinical parameters over patient-relevant endpoints in the development of study designs. Furthermore, it is anticipated that a significant portion of the identified studies will exhibit relatively poor methodological quality and provide only limited generalisable results.

**Systematic review registration:**

This study is registered within PROSPERO (CRD42023412156).

## Background

Artificial intelligence (AI) is a broad term referring to the field of computer science that develops algorithms mimicking human cognitive functions such as learning, perception, problem-solving and decision-making. AI encompasses various approaches, including machine learning (ML) and deep learning. It comprises a range of technologies and techniques, including algorithmic decision-making (ADM) ([9]: 1). ADM refers to the process of using these algorithms to gather, process, model and use input data to make or support decisions. Feedback from these decisions can then be used for improving the system ([2]: 612). An ADM can take various forms depending on how it is framed and presented to the user or decision subject. It can be a simple algorithm that has been known and used for decades, such as classification trees [37], or a more complex system like a recommender or AI that can provide recommendations to human decision-makers, nudge its users in a certain direction or perform fully automated decision-making processes without human involvement ([2]: 613). We specify AI-related algorithmic decision-making systems (AI-related ADM) as decision support systems that either apply AI (relying on ML models) or have been developed with the help of AI.

Recent advances in AI have resulted in the development of more complex and sophisticated systems that can outperform humans in certain tasks. For example, in the field of computer vision, systems like DeepMind’s AlphaFold have revolutionised protein structure prediction, solving a decades-old challenge in biology by accurately predicting 3D protein structures [18]. Additionally, AI innovations have transformed financial services, with machine learning models now being used to predict market trends, optimise trading strategies and enhance fraud detection [12]. Furthermore, generative AI has demonstrated remarkable capabilities in generating human-like text and performing a wide range of language-related tasks with unprecedented accuracy [13]. Recently, ChatGPT was evaluated for its clinical reasoning ability by testing its performance on questions from the United States Medical Licensing Examination, where it scored at or near the passing threshold on all three exams without any special training or reinforcement [21].

These advances in AI seem to have enormous potential to transform many different fields and industries, which begs the question: will AI do so in healthcare?

In clinical trials, AI systems have already shown potential to help clinicians make better diagnoses [3, 22], help personalise medicine and monitor patient care [6, 16] and contribute to drug development [7]. However, successful application in practice is limited ([30]: 77) and potential issues that may be responsible for this gap between research and practice should be revealed by our work.

By searching PubMed for the term ‘artificial intelligence’, we found over 2000 systematic reviews and meta-analyses published in the last 10 years, with a yearly increasing trend. These include several reviews conducted in the area of AI in healthcare that provide an overview of the current state of AI technologies in specific clinical areas, including AI systems for breast cancer diagnosis in screening programmes [8], ovarian cancer [38], early detection of skin cancer [17], COVID-19 and other pneumonia [15], prediction of preterm birth [1] or diabetes management [19]. Other reviews have focused on comparing clinicians and AI systems in terms of their performance to show their capabilities in a clinical setting [24, 27, 34].

Although these reviews are crucial to the further development of AI systems, they offer little insight into whether patients actually benefit from their use by medical professionals. Indeed, these studies focus on the analytical performance of these systems, rather than on healthcare-related metrics. In most of the studies mentioned here, the underlying algorithms have been evaluated using a variety of parameters, such as the F1 score for error classification, balanced accuracy, false positive rate and area under the receiver operating characteristic curve (AUROC). However, measures of a system’s accuracy often provide non-replicable results ([25]: 4), do not necessarily indicate clinical efficiency ([20]: 1), AUROC does not necessarily indicate clinical applicability ([10]: 935) and in fact, none of these measures reflects beneficial change in patient care ([4]: 1727, [33]: 1).

To summarise, as with any other new technology introduced into healthcare, the clinical effectiveness and safety of AI compared to the standard of care must be evaluated through properly designed studies to ensure patient safety and maximise benefits while minimising any unintended harm ([31]: 328). Therefore, a critical analysis of patient-relevant outcomes is needed, especially the benefits and harms of decisions informed by or made by AI systems.

To this end, this review goes beyond previous studies in several ways. First, we study clinical AI systems that enable algorithmic decision-making (AI-related ADM) in general and therefore do not limit ourselves to selected clinical problems. In particular, we focus on machine learning systems that infer rules from observations. Although we omit rule-based systems, we apply the term AI throughout our work because it is often incorrectly and redundantly used for ML and deep learning in the literature we study. Second, we focus on studies that report patient-relevant outcomes that, according to German Institute for Quality and Efficiency in Healthcare ([14]: 44), describe how patients feel, how they can perform their functions and activities or if they survive. These may include, for example, mortality, morbidity (with regard to complaints and complications), length of hospital stay, readmission, time to intervention and health-related quality of life. Third, we focus only on studies that compare medical professionals supported by AI-related ADM systems with medical professionals without AI-related ADM systems (standard care). By doing so, this review provides an overview of the current literature on clinical AI-related ADM systems, summarises the empirical evidence on their benefits and harms for patients and highlights research gaps that need to be addressed in future studies.

## Objectives

The aim of this review is to systematically assess the current evidence on patient-relevant benefits and harms of ADM systems which are developed or used with AI (AI-related ADM) to support medical professionals compared to medical professionals without this support (standard care).

1. Are there studies that compare patient-relevant effectiveness of AI-related ADM for medical professionals compared to medical professionals without AI-related ADM?
2. Do these studies show adequate methodological quality and are their findings generalisable?
3. Can AI-related ADM systems help medical professionals to make better decisions in terms of benefits and harms for patients?

## Methods/design

In accordance with the Preferred Reporting Items for Systematic Review and Meta-Analysis Protocols (PRISMA-P) statement [26], the study protocol for this systematic is registered on the International Prospective Register of Systematic Reviews (PROSPERO) database (CRD42023412156). If necessary, post-registration changes to the protocol will be detailed under the PROSPERO record with an accompanying rationale.

We will follow the Preferred Reporting Items for Systematic Reviews and Meta-Analyses (PRISMA) statement [29] and the Methodological Expectations of Cochrane Intervention Reviews (MECIR) standards [11].

### Searches

We will search systematically using English free text terms in title/abstract, Medical Subject Headings (MeSH) terms and Embase Subject Headings (Emtree) fields for various forms of keywords related to ‘artificial intelligence’ and relevant subcategories of computer generated and processed decision-making algorithms, ‘medical professionals’ and keywords describing effectiveness parameters and outcomes as well as preferred study types. Based on the block building approach, keywords and terms are combined using the Boolean operators AND and OR and progressively checked for relevant hits.

### Databases to be used for searches

MEDLINE and PubMed (via PubMed), Embase (via Elsevier) and Institute of Electrical and Electronics Engineers (IEEE) Xplore will be searched for peer-reviewed articles as well as ClinicalTrials.gov and ICTRP (via CENTRAL) for ongoing trials and protocols.

To reduce potential publication bias, additional studies will be identified by contacting authors of included studies, contacting experts in the field and through reference lists of relevant studies. Grey literature searches will be conducted in Google Scholar. For this purpose, the keywords used in the systematic search will be used in different combinations, as well as their German equivalents. Google Scholar will be searched up to the 10th hit page. The detailed search strategy for each database will be reported under the PROSPERO record once the searches have been conducted.

### Search strategy

We developed our search strategy using the PICOS scheme (Table 1).

**Table 1:** PICOS scheme

**Participants**	**Intervention**	**Control**	**Outcome**	**Study type**
Human patients without restriction in age or sex	Medical professionals supported by an AI-related ADM system applied to a clinical problem	Medical professionals applied to a clinical problem, without support by an AI-related ADM system (standard care)	Patient-relevant benefits and harms	Interventional and observational studies

While doing preliminary searches for basic literature in MEDLINE and PubMed (via PubMed), we noticed that study conductors from different scientific fields (e.g. computer scientists) used different terms for the intervention outcomes we were looking for. In addition, some studies were not indexed appropriately in PubMed, which complicated our initial search strategy. To carry out the search strategy, we have created and tested the blocks consecutively to gather the best results from each block, expanding and narrowing the search strategy. To assess the right direction of the search strategy, we have used fundamental literature, such as Choudhury and Asan [5], Park et al. [31] and Nagendran et al. [27] as test sets, making sure the results of our search had common ground with these studies.

The resulting search string for MEDLINE and PubMed in the individual blocks can be found in Table 2 and describes the basis for other databases.

**Table 2:** Search string blocks for MEDLINE and PubMed (via PubMed)

Block 1, artificial intelligence	((“artificial intelligence”[MeSH Terms] OR “artificial intelligence”[Title/Abstract] OR “artificial-intelligence”[Title/Abstract] OR “machine learning”[Title/Abstract] OR “machine-learning”[Title/Abstract] OR “hierarchical learning”[Title/Abstract] OR “computational intelligence”[Title/Abstract] OR “machine intelligence”[Title/Abstract] OR “computer reasoning”[Title/Abstract] OR “deep learning”[Title/Abstract] OR “supervised learning”[Title/Abstract] OR “unsupervised learning”[Title/Abstract] OR “reinforcement learning”[Title/Abstract] OR “representation learning”[Title/Abstract] OR “natural language processing”[Title/Abstract] OR “large language model*”[Title/Abstract] OR “generative model*”[Title/Abstract] OR “representation learning”[Title/Abstract] OR (“knowledge acquisition”[Title/Abstract] AND “computer”[Title/Abstract]) OR (“knowledge representation”[Title/Abstract] AND “computer”[Title/Abstract]) OR “image recognition”[Title/Abstract] OR “machine vision”[Title/Abstract] OR “computer vision”[Title/Abstract] OR “algorithmic decision”[Title/Abstract]))
Block 2, medical professionals	AND (“expert”[Title/Abstract] OR “experts”[Title/Abstract] OR “medical professional”[Title/Abstract] OR “medical professionals”[Title/Abstract] OR “medical doctor*”[Title/Abstract] OR “physician*”[Title/Abstract] OR “clinician*”[Title/Abstract] OR “general practitioner*”[Title/Abstract] OR “health care professional”[Title/Abstract] OR “health care professionals”[Title/Abstract] OR “healthcare professional”[Title/Abstract] OR “healthcare professionals”[Title/Abstract] OR “nurse”[Title/Abstract] OR “nurses”[Title/Abstract] OR (“therapist”[Title/Abstract] OR “therapists”[Title/Abstract]) OR (“health”[Title/Abstract] AND “alert system”[Title/Abstract]) OR (“medical”[Title/Abstract] AND “alert system”[Title/Abstract]) OR (“practice”[Title/Abstract] AND “alert system”[Title/Abstract]) OR (“hospital”[Title/Abstract] AND “alert system”[Title/Abstract]) OR (“clinic*”[Title/Abstract] AND “alert system”[Title/Abstract]) OR (“health”[Title/Abstract] AND “decision support”[Title/Abstract]) OR (“medical”[Title/Abstract] AND “decision support”[Title/Abstract]) OR (“practice”[Title/Abstract] AND “decision support”[Title/Abstract]) OR (“hospital”[Title/Abstract] AND “decision support”[Title/Abstract]) OR (“clinic*”[Title/Abstract] AND “decision support”[Title/Abstract]) OR (“health”[Title/Abstract] AND “warning system”[Title/Abstract]) OR (“medical”[Title/Abstract] AND “warning system”[Title/Abstract]) OR (“practice”[Title/Abstract] AND “warning system”[Title/Abstract]) OR (“clinic*”[Title/Abstract] AND “warning system”[Title/Abstract]))
Block 3, outcomes	AND (“effectiveness”[Title/Abstract] OR “effectivity”[Title/Abstract] OR “benefit”[Title/Abstract] OR “benefits”[Title/Abstract] OR “harm”[Title/Abstract] OR “harms”[Title/Abstract] OR “adverse event*”[Title/Abstract] OR “mortality”[Title/Abstract] OR “morbidity”[Title/Abstract] OR “length of hospital stay”[Title/Abstract] OR “readmission”[Title/Abstract] OR “time to intervention”[Title/Abstract] OR “health-related quality of life”[Title/Abstract] OR “endpoint*”[Title/Abstract] OR “outcome*”[Title/Abstract])
Block 4, study types	AND (“randomised” OR “randomized” OR “RCT” OR “clinical trial*” OR “cohort” OR “observational study” OR “observational design*” OR “case–control” OR “experiment*” OR “retrospective study” OR “retrospective design*” OR “prospective study” OR “prospective design*” OR “non-inferiority” OR “phase* study” OR “intervention study” OR “diagnostic study” OR “pre-post study” OR “pre post study” OR “pre-post design” OR “pre post design”)
Filter	AND (humans[filter]) AND (y_10[filter])

#### Types of studies to be included

For the systematic search, peer-reviewed interventional and observational studies published in German or English 10 years retrospectively from the date of the search will be considered. For the search of grey literature, scientific reports published in German or English 10 years retrospectively from the date of the search will be considered. To extract potentially relevant studies from (systematic) reviews and meta-analyses, secondary studies will be gathered and screened. However, secondary studies will not be included in the synthesis.

In contrast to studies of effectiveness and safety, pure efficacy studies (e.g. focusing on algorithms accuracy) will be excluded as these outcomes are not directly relevant for patients. Patient-relevant outcomes will be defined according to the IQEHC method paper [14]. In addition, studies that used AI systems beyond our scope, such as robotics (systems that support the implementation of decisions), will be excluded. Editorials, commentaries, letters and other informal publication types will be excluded as well.

We will provide a list of all references screened in full text including exclusion reasons in the appendix of the final study.

#### Participants

Our study is focusing on human patients without restriction of age or sex. Therefore, the input data for the algorithms must include real human data gathered either during routine care and saved for use in research or generated specifically for the individual study.

#### Intervention

Out study is focusing on medical professionals utilising an AI-related ADM system to address a clinical problem.

In our working definition, a medical professional is a qualified individual who has the authority to perform necessary medical procedures within their professional scope of practice. Their goal is to improve, maintain or restore the health of individuals by examining, diagnosing, prognosticating and/or treating clinical problems. This may include medical doctors, registered nurses and other medical professionals. Clinical problems can encompass illnesses, injuries and physical or mental disorders, among other conditions.

In our working definition, an AI-related ADM system is a clinical decision support system that either applies AI in the sense of machine learning (ML, excluding rule-based systems) or has been developed with the help of ML. Clinical decision support models without any involvement of AI will be excluded.

#### Control

Medical professionals, as described in the working definition, are addressing a clinical problem without the support of an AI-related ADM system (standard care).

#### Outcomes

Patient-relevant benefits and harms, according to the IQEHC method paper [14], are gathered. These may include, for example, mortality, morbidity (with regard to complaints and complications), length of hospital stay, readmission, time to intervention and health-related quality of life.

#### Study types

We will collect both interventional and observational studies, which may encompass randomised controlled trials, cohort studies, case–control studies, randomised surveys, retrospective and prospective studies and phase studies, as well as non-inferiority or diagnostic studies.

### Data extraction

Records arising from the literature search will be stored in the citation manager Citavi 6 (c) by Swiss Academic Software. After removing duplicates, two reviewers will independently review all titles and abstracts via the browser application Rayyan [28]. Studies potentially meeting the inclusion criteria will then be screened in full text independently by two reviewers using Citavi 6 (c). Disagreements over eligibility of studies will be discussed and, if necessary, resolved by a third reviewer. Authors of the included studies will be contacted if clarification of their data or study methods is required. The PRISMA 2020 flow diagram [29] will be used to keep the study selection process transparent.

Using a standardised data collection form, two reviewers will extract data independently from the included studies and will compare them for discrepancies. Missing data will be requested from study authors. Extracted data will include country of conduction, setting, study design, observational period, patient-relevant outcomes, intervention, comparator, characteristics of patient and medical professional populations and characteristics of the used algorithm. Additionally, studies will be classified by type of system, medical specialty or clinical area, prediction or classification goal of the AI-related ADM, supported decision, investigated benefits and harms, private or public study funding, applicable regulation (e.g. FDA, MDR), medical device classification (based on the risk and nature of the product) and whether the product is commercially available in its respective class (Table 3).

**Table 3:** Study data to be extracted

**Table/item**	**Example**
**Study characteristics**	
Reference, registration	Meier, 2022
Country of conduction	Germany
Setting	Hospital
Study design	RCT
Observation duration	January 2017 until September 2018
Medical specialty	Intensive care unit (ICU)
Prediction/classification goal of AI-related ADM	Sepsis
Patient-relevant outcome	Mortality, length of hospital stay
Intervention procedure/ instrument	ICU bedside monitors with recommender
Comparison procedure/ instrument	ICU bedside monitors without recommender
Study funding	No funding
**Characteristics of the evaluation population**	
Patient population	
Inclusion criteria	Participants age over 18 under 64
Exclusion criteria	Pre-existing septic shock
Mean age (SD)	49.8 (1.55)
Population total (share of sex in %)	*N* = 75 (*n* = 30 females)
Medical professional population	
Inclusion criteria	ICU physician, trained in used AI-related ADM system
Exclusion criteria	Physician at ICU for less than 2 years
Mean age (SD)	45.0 (3.5)
Population total (share of sex in %)	*N* = 6 (*n* = 3 females)
**Characteristics of used algorithm**	
Algorithm name	ResNet-18
Algorithm architecture	Convolutional neural network (CNN)
Data source	In-house digital medical records, monitoring data
Development	Laboratory and health metrics (HR, RR, SpO2, etc.) of *n* = 677 cases (*n* = 220 females)
Validation	Internal: random split sample, external: no
Applicable regulation	MDR
Medical device classification	Class I device: low risk, non-invasive
Commercial availability	No
**Risk of bias assessment (RoB 2/ROBINS-I)**	
** Reporting assessment (CONSORT-AI)**	
**Study results**	
Supported decision	Initiation of life-saving measures, hospital discharge
Patient benefit (effect size)	Reduction length of hospital stay: 2.3 days Mortality rate reduction: 12/100 patients Reduction length of stay ICU: 0 days
Patient harm (effect size)	Not reported
Other effects	Not reported
**Assessment for clinical use**	
Implementation status	Not implemented
Author’s restrictions on clinical use	System requires more training and testing
Author’s recommendation on clinical use	Not mentioned

### Risk of *bias* and quality assessment

Risk of bias will be assessed by using the revised Cochrane risk-of-bias tool for randomised trials (RoB 2) [36] and the risk-of-bias in non-randomised studies for interventions (ROBINS-I) tool [35]. Disagreements between the authors over the risk of bias in the included studies will be resolved by discussion or with involvement of a third author if necessary. Transparent reporting of the included studies will be assessed trough the Consolidated Standards of Reporting Trials interventions involving Artificial Intelligence (CONSORT-AI) extension by Liu et al. [23]. The CONSORT-AI extension includes 14 new items that were considered sufficiently important for AI interventions to be routinely reported in addition to the core CONSORT items by Schulz et al. [32]. CONSORT-AI aims to improve the transparency and completeness in reporting clinical trials for AI interventions. It will assist to understand, interpret and critically appraise the quality of clinical trial design and risk of bias in the reported outcomes. We will assess studies conducted prior to the introduction of the CONSORT-AI guidelines in 2020 against these standards where possible. Although these studies may not fully meet the new criteria, application of the guidelines may still identify potential reporting gaps and ensure a consistent assessment framework across studies. We will discuss limitations related to this retrospective requirement to ensure a balanced and comprehensive analysis.

### Data synthesis

Given the expected likelihood of heterogeneity between studies in the different medical specialties in terms of outcome measures, study designs and interventions, we do not know if performing a meta-analysis will be possible. However, a systematic narrative synthesis will be provided of the results with an overview of the relevant effects for the outcomes, with information presented in the text and tables to summarise and explain the characteristics and findings of the included studies. We will analyse the geographic distribution, study settings and medical specialties of the included studies. Additionally, we will examine funding sources and conduct a detailed risk of bias assessment. Compliance with reporting standards, such as CONSORT-AI and TRIPOD-AI, will be evaluated. We also plan to analyse patient demographics, including age, sex and race/ethnicity, as well as the involvement and training of medical professionals. ADM systems will be categorised into applicable regulation (e.g. FDA, MDR), medical device classification (based on the risk and nature of the product) and whether the product is commercially available in its respective class. Outcome analyses will focus on assessing both benefits and harms. Furthermore, we will analyse the validation of algorithms, considering both internal and external validation, and review the data availability statements to evaluate the accessibility of data used for algorithm development. Studies with an unclear or high risk of bias are not excluded to avoid potential selection bias and to ensure that valuable findings, particularly in emerging areas, are not lost. By including them, but clearly acknowledging and discussing their limitations, we aim to provide a more comprehensive overview of the available evidence. For this reason, our narrative synthesis emphasises the qualitative aspects of the data and focuses on identifying and describing trends, patterns and inconsistencies in the studies, rather than attempting to quantify effect sizes. This is consistent with the approach of recent reviews examining the methodological quality of machine learning systems in clinical settings (e.g. [27]).

## Discussion

It is to be expected that there is a significant lack of suitable studies comparing healthcare professionals with and without AI-related ADM systems regarding patient-relevant outcomes. It is assumed that this is due to, first, the lack of approval regulations for AI systems, second, the prioritisation of technical and clinical parameters over patient-relevant outcomes in the development of study designs and, third, the prioritisation of AI for supporting clinical processes (e.g. administration). In addition, it is to be expected that a large proportion of the studies to be identified are of rather poor methodological quality and provide results that are rather difficult to generalise. Although reporting guidelines such as the Consolidated Standards of Reporting Trials (CONSORT) statement [32] are well-known and widely used in medical and public health research, they do not necessarily correspond to the novel protocol and study designs that are relevant for the assessment of the research questions relevant here. The extension of the Reporting Guidelines for Clinical Study Reports of Interventions Using Artificial Intelligence (CONSORT-AI) [23] may fill the gap but this guideline is relatively new and not necessarily always applied.

## Data Availability

Not applicable.

## Abbreviations

ADM: Algorithmic decision-making
AI: Artificial intelligence
AUROC: Area under the receiver operating characteristic curve
CENTRAL: Cochrane Central Register of Controlled Trials
CNN: Convolutional neural network
CONSORT: Consolidated Standards of Reporting Trials
CONSORT-AI: Consolidated Standards of Reporting Trials for Artificial Intelligence
CRD: Centre for Reviews and Dissemination
Emtree: Embase Subject Headings
HR: Heart rate
ICU: Intensive care unit
IEEE: Institute of Electrical and Electronics Engineers
IQEHC: German Institute for Quality and Efficiency in Healthcare
MECIR: Methodological Expectations of Cochrane Intervention Reviews
MeSH: Medical Subject Headings
ML: Machine learning
nRCT: Non-randomised controlled trial
PICO: Participants, Intervention, Control, Outcome
PRISMA: Preferred Reporting Items for Systematic Review and Meta-Analysis
PRISMA-P: Preferred Reporting Items for Systematic Review and Meta-Analysis Protocols
PROSPERO: International Prospective Register of Systematic Reviews
RCT: Randomised controlled trial
ResNet-18: A convolutional neural network that is 18 layers deep
RoB 2: Revised Cochrane risk-of-bias tool for randomised trials
ROBINS-I: Risk-of-bias in non-randomised studies for interventions
RR: Respiratory rate
SD: Standard deviation
SpO2: Oxygen saturation
SRQR: Standards for Reporting Qualitative Research
